# Supplementary material for: Astroglial Connexins Inactivation Increases Relapse of Depressive-like Phenotype after Antidepressant Withdrawal
Source: Int J Mol Sci. 2022 Oct 30;23(21):13227. doi: 10.3390/ijms232113227 (PMC9656718; doi:10.3390/ijms232113227)

Supplemental Figure S1: Mice's fur is degraded after a chronic exposure to corticosterone. Corticosterone was delivered in drinking water and the state of fur was evaluated every week for ten weeks (A). On week 10, the score is significantly higher in the CORT treated animal compared to VEH treated indicating a depressive effect of the chronic exposure to CORT (B). Student t-test: ***p<0.001: statistically different as indicated.

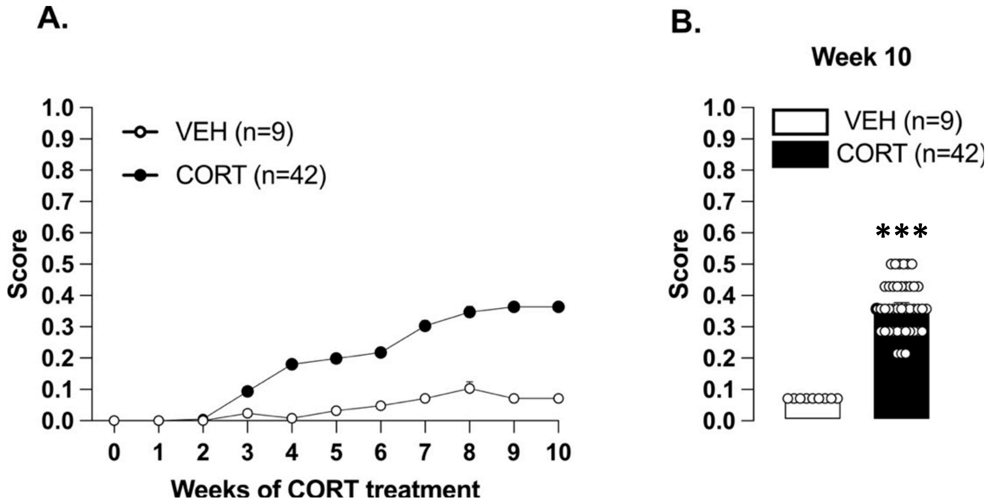

Methods: Biochemical Assays. Blood sampling was performed on mice that were not tested in behavioral procedures to avoid the influence of acute stress on the analysis. For the assessment of blood corticosterone levels, blood from CORT mice treated with the Cx blocker carbenoxolone (CBX) or its vehicle was drawn from the tip of the tail vein at 9 am. Corticosterone was quantified using an ELISA kit (Arbor Assays).

Supplemental Figure S2: Effect of the Connexins blocker carbenoxolone on plasma corticosterone levels. Data are means ± SEM of plasma corticosterone levels (ng/ml) after chronic treatment with carbenoxolone (CBX) or its vehicle in corticosterone (CORT) exposed mice. blood corticosterone was measured using an ELISA kit. Student t-test: ns: not statistically different.

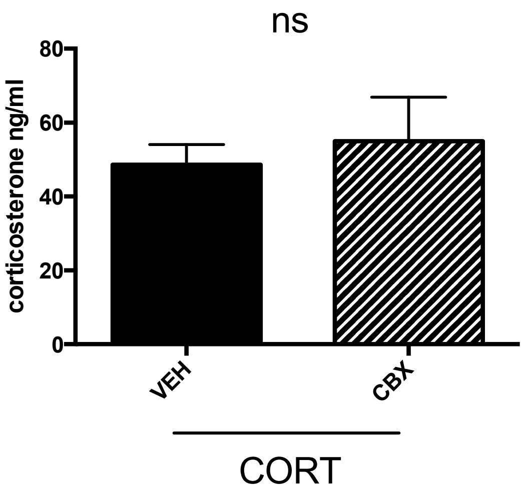

Supplement: Supplementary file 1 [file ijms-23-13227-s001.zip › ijms-1988105-supplementary.pdf]
